# The conceptualisation and operationalisation of ‘marketing’ in public health research: a review of reviews focused on food marketing using principles from critical interpretive synthesis

**DOI:** 10.1186/s12889-023-16293-4

**Published:** 2023-07-24

**Authors:** Hannah Forde, Yanaina Chavez-Ugalde, Rebecca A Jones, Kate Garrott, Prasanti Alekhya Kotta, Felix Greaves, Victoria Targett, Martin White, Jean Adams

**Affiliations:** 1 Nuffield Department of Primary Care Health Sciences, Radcliffe Observatory Quarter, Woodstock Road, Oxford, OX2 6GG UK; 2 MRC Epidemiology Unit, Institute of Metabolic Science, University of Cambridge School of Clinical Medicine, Cambridge Biomedical Campus, Box 285, Cambridge, CB2 0QQ UK; 3 Bristol Medical School, University of Bristol, First Floor, 5 Tyndall Avenue, Bristol, BS8 1UD UK; 4 Institute of Metabolic Science, University of Cambridge School of Clinical Medicine, Cambridge Biomedical Campus, Box 285, Cambridge, CB2 0QQ UK; 5 Department of Medicine, Baylor College of Medicine, Houston, TX USA; 6 Department of Primary Care and Public Health, Imperial College London, Charing Cross Hospital, London, W6 8RP UK; 7 National Institute for Health and Care Excellence, 2 Redman Place, London, E20 1JQ UK; 8 Department of Health and Social Care, London, UK; 9 Public Health England, London, UK

**Keywords:** Food, Diet, Marketing, Public health, Review, Critical interpretive synthesis.

## Abstract

**Background:**

Extensive public health research reports the nature, scope and effects of various marketing activities used by food and drinks companies to support the sale of their products. Such literature informs the regulation of food marketing that encourages unhealthy eating behaviours and poor diet-related health outcomes. However, it is not clear whether this literature consistently conceptualises and applies marketing, which could in turn influence the approach and efficacy of policies to regulate food marketing. We aimed to understand the conceptualisation and operationalisation of marketing in public health research of food marketing, eventually focusing on the conceptualisation of integrated marketing.

**Methods:**

We conducted a review of reviews that drew on scoping review methods and applied principles of critical interpretive synthesis. Five databases of peer-reviewed literature and websites of relevant organisations were searched in June – August 2020. Articles were screened against inclusion criteria to identify reviews examining food marketing in a health context. Informative text segments from included articles were coded using NVivo. Codes were grouped into synthetic constructs and a synthesising argument.

**Results:**

After screening against inclusion criteria, 60 publications were eligible for inclusion. Informative text segments from 24 publications were coded, after which no new codes were identified. Our synthesising argument was that the understanding of integrated marketing appeared inconsistent across publications, such as by differences in use of underlying conceptual frameworks and in the application of terms such as marketing strategy and tactics.

**Conclusions:**

Using our synthesising argument, we suggest ways to improve the future study of food marketing in public health research, for example by using in-depth case studies to understand the integrated operation and effect of multi-component marketing strategies. Improving conceptual clarity in the study of food marketing in public health research has the potential to inform policy that is more reflective of the true nature of marketing, and thus more effective in combating food marketing effects and protecting public health.

**Protocol registration:**

The review protocol was made publicly available on Open Science Framework prior to the start of the study (DOI: 10.17605/OSF.IO/VSJCW).

**Supplementary Information:**

The online version contains supplementary material available at 10.1186/s12889-023-16293-4.

## Background

The food and non-alcoholic beverage industry use marketing to increase sales and maximise company profits [1]. As many products produced by these industries are not aligned with dietary guidance, marketing can contribute to the development of non-communicable diseases (NCDs) [2].

Marketing involves a range of practices that includes those related to product development, pricing, promotion, branding and placement. In business literature, marketing is defined as “the activity, set of institutions and processes for creating, communicating, delivering and exchanging offerings that have value for customers, clients, partners, and society at large” [3], coordinated by an integrated marketing strategy. In the public health literature, marketing is often conceptualised using the four (five, six or even seven) Ps of the marketing mix: price, product, promotion, placement [4, 5] (people, process and physical evidence) [6]. Such conceptualisation may not emphasise the importance of these elements being part of an integrated strategy [7].

Regulating some aspects marketing of less healthy food and non-alcoholic beverages (referred to as ‘food’ hereafter) may help contribute to NCD prevention. However, the effectiveness of such regulatory policies may be undermined by food companies adapting their marketing in response, particularly by expanding unregulated forms of marketing [8]. This phenomenon may be referred to as the “balloon effect” [9, 10], whereby marketing strategies adapt to a ‘squeeze’ in one part of the system caused by a new regulatory environment, by expanding in other parts to maintain the overall equilibrium (i.e., an expected level of sales or profits) of the system [8, 11]. For example, taxes on sugary drinks may lead to increased prices and subsequent increases in advertising to protect sales and so minimising any effects on public health [12]. The responsive process described by the balloon effect reflects the strategic integration of marketing activities, and has resulted in support for simultaneous regulation of multiple aspects of food marketing [8, 13]. For example, the amended Chilean sugary drinks tax (2014) [14] was coupled with a Food Labelling and Advertising Law (2016) [15] leading to a package of measures focused on the price, advertising and placement of less healthy foods.

Whilst the Chilean approach at least partly reflects the integrated nature of marketing as understood from a business literature perspective, few other countries have achieved such a co-ordinated response to less healthy food marketing [16]. One possible reason for this is a limited or inconsistent conceptualisation and operationalisation of marketing in the public health literature and, by extension, public health policymaking. There is abundant public health research assessing the nature, scope and effects of individual components of food marketing (e.g. advertising) and many reviews of these [17–19], yet it is not clear how much of this literature reflects an integrated understanding of marketing. We addressed this question using a review that drew on scoping review methods.

Most scoping reviews aim to provide an aggregated overview of a body of literature [20]. However, aggregative approaches are often insufficient to provide a theoretical account that is neither too abstract nor too specific to understand a concept [21, 22]. Interpretive synthesis reviews are better for induction and interpretation that develops concepts grounded in the studies reviewed, but most interpretive synthesis methods are designed to synthesise qualitative research [21]. Critical interpretive synthesis (CIS) is one approach to synthesising research regardless of study design [21]. CIS permits the reviewer to critique the treatment and underpinning assumptions of the phenomenon of interest [21], producing a “mid-range” theoretical account of the evidence that has both empirical applicability and explanatory scope. CIS recognises that it may be neither possible nor desirable to specify the precise research questions of a review at the outset [21].

In this review we set out with the broad aim of understanding the conceptualisation and operationalisation of “marketing” in public health research on food marketing. As analysis progressed, our aim evolved to focus on the conceptualisation of ‘integrated marketing’ in order to understand whether the public health literature sufficiently appreciates the integrated nature of marketing as described in the business literature. We drew on scoping review methods and employed principles of CIS to meet this aim.

## Methods

We conducted a review of reviews, adapting Arksey and O’Malley’s six-step scoping review framework to apply principles of CIS [23–26]. These six steps are (1) identifying the research question, (2) identifying relevant studies, (3) study selection, (4) charting the data, (5) collating, summarising and reporting the results, and (6) consulting with key stakeholders. We applied principles from CIS to steps [3]–[5], entailing back-and-forth movement and coding in order to develop a synthesising argument. As previously [27], the review was pragmatic and pluralistic in that we included a range of literature from different perspectives and using different methods, and decisions about inclusion were informed by judgements of relevance. The review was made publicly available on Open Science Framework (DOI: 10.17605/OSF.IO/VSJCW), and is reported in line with the Preferred Reporting Items for Systematic Reviews and Meta-analyses extension for Scoping Reviews (PRISMA-ScR) (Additional file 1) [28].

### Steps (1) and (2): search methods for the identification of studies

Preliminary searches guided the development of our research question and search strategy. Though CIS assumes the topic under study to have diffuse boundaries [21], the existence of distinct definitions of marketing in non-public health literature guided our search (Table 1). The resultant eligibility criteria are less bounded than those of an aggregative review [21] (Table 2). We included reviews that explored any form of commercially-derived, or mimicking commercially-derived, food marketing, even if not explicitly described as such by authors. As this evidence base is vast, we restricted our search to reviews (as defined by the authors of publications) that were supported by a description of a method for searching the literature. We only included reviews that explored food marketing in the explicit context of health. Searches were limited to literature published in English because conceptualisations could differ by language, and nuances might be lost in translation. Literature published before 2006 was excluded since a transformative World Health Organization technical paper on food promotion was published in 2006 [19].

**Table 1:** Relevant definitions informing the research question and search method

Term	Definition
Marketing	“the activity, set of institutions and processes for creating, communicating, delivering and exchanging offerings that have value for customers, clients, partners, and society at large” [3]
Marketing mix (4Ps)	Product, price, promotion, place [5]
Marketing strategy	“An over-riding directional concept that sets out the planned path” [29] “Lays out target markets and the value proposition that will be offered based on an analysis of the best market opportunities” [7]
Integrated marketing communications	“A strategic business process used to plan, develop, execute and evaluate coordinated, measurable, persuasive brand communication programmes over time with consumers, customers, prospects, and other targeted, relevant external and internal audiences.” [30, 31]

**Table 2:** Eligibility criteria

Category	Inclusion criteria	Exclusion criteria
Population type	Any human population	Animal populations
Year of publication	In or after 2006	Before 2006
Publication type	Any author-defined review of published evidence (i.e. must include ‘review’ as a self-descriptor) and provide a specified method for searching, retrieval and analysis of published material.	Not an author-defined review of published evidence (i.e. does not say ‘review’) or does not have a specified review method.
Country	Any country	N/A
Language	Published in English	Not published in English
Focus on marketing	Relates to any form of commercially-derived, or mimicking commercially-derived, marketing (e.g. promotion, product, placement, price…etc.), across any possible medium (e.g. internet, TV, retail environment).	Any form of marketing that does not derive from a commercial source or mimic that provided by a commercial source (e.g. social marketing, public health marketing, more general effect/ behaviour of media use).
Food or drink related	Relates to marketing of any food/non-alcoholic drink	Marketing not specific to food/non-alcoholic drinks (e.g. for alcohol, tobacco or other products).
Health context	Review must be in the explicit context of health (e.g. must refer to a health-related outcome or interest)	Review not in the explicit context of health (i.e. does not refer to a health-related outcome or interest).

Academic librarians advised on the search strategy. We conducted title and abstract searches in Ovid MEDLINE, Ovid Embase, APA PsycInfo (EBSCO), Web of Science Core Collection (no restriction to indices) and Cochrane Library on 8th July 2020 using search terms relating to marketing, food and non-alcoholic beverages, health, and reviews, and index terms where appropriate (full search in Additional File 2). We conducted grey literature searches between June – August 2020 [32], by purposively selecting relevant organisations, informed by the research team’s expertise, and searched their websites for permutations of the terms “food”, “marketing”, “advertising” and “promotion”. We included organisations based in the UK (Food Foundation, Obesity Health Alliance, Public Health England, Sustain, UK Health Forum, Cancer Policy Research Centre, Institute for Social Marketing and Health), the USA (Centre for Science in the Public Interest, UConn Rudd Center), and a global organisation (World Health Organisation). We subsumed publications identified in the grey literature search at the full text screening stage.

All records from the database searches were retrieved, stored and de-duplicated in Endnote [33], a reference management software, and Covidence [34], a web-based platform that streamlines the production of systematic reviews. We used both Endnote and Covidence to maximise the identification of duplicate records, since they each identify slightly different duplicates. HF, YCU, RAJ, KG, PAK, and JA piloted screening for a sample of 200 records, discussing discrepancies and refining the criteria. We improved rigour by conducting simultaneous independent screening of each record by two authors (HF screened all publications; second screening was divided across YCU, RAJ, KG, and PAK). Records which both reviewers agreed did not meet the eligibility criteria were excluded. The full texts of remaining records were retrieved and sources from the grey literature searches added. All records with conflicting decisions between screeners at title and abstract screening advanced to full text review to minimise risk of excluding potentially relevant texts.

The simultaneous independent screening process by two reviewers was repeated for full texts, with disagreements resolved in discussion, arbitrated by JA where necessary. We used a hierarchy of reasons for exclusion with the highest reason selected by either screener retained. As is often the case for scoping reviews, we did not assess credibility since the aim was to describe practice rather than assess the certainty of results. Instead, judgements of relevance were made during synthesis based on researchers’ perceptions of relevance rather than a formal quality assessment tool [21, 24, 35].

### Steps (3) to (5): sampling, extraction and critical interpretive synthesis (CIS)

The nature of CIS required iterative movement between steps [3–5] of Arksey and O’Malley’s framework. Using CIS, researchers develop synthetic constructs. These are third order constructs that result from an interpretation of the whole of the evidence to unify several disparate aspects of a phenomenon in an explanatory way [21]. Though definitions of first, second and third order constructs vary, we drew on existing working definitions: first order constructs were narratives of marketing directly in the texts, second order constructs were our views and interpretations of these narratives, and third order constructs were our interpretations of the second order constructs [36]. Thus, our implicit or explicit understanding of marketing concepts inevitably influenced our interpretation of texts and resultant findings.

Using synthetic constructs supplemented by second order constructs and possibly other evidence, CIS produces a synthesising argument, which integrates evidence into a coherent theoretical framework in order to provide a generalisable way of understanding a phenomenon [21]. Blending sampling, extraction and analysis alongside constant comparison with the data is an expectation of CIS [21].

To conduct CIS here, first, metadata for all included studies, such as publication information and the marketing component under study, were extracted into a Microsoft Excel spreadsheet. Our perception of ‘thickness’ was also recorded. Here, thickness referred to the detail and volume of description about marketing that was included in the text [37–39]. As there is strong evidence of bias in studies of the relationship between food and health where the food industry funds – or is involved in – the research [40], we also recorded whether authors reported conflicts of interest (e.g. funding) related to the food and drinks industry. As has been the case for other CIS reviews [21], we found a traditional data extraction process driven by *a-priori* categories and closed questions ill-suited to our aims and the diverse body of included literature. Instead, we selected studies, extracted data, and analysed inductively, applying methods from other CIS reviews that drew on principles of grounded theory [21].

We set out with no target minimum sample of texts from which we sought to extract and analyse. Instead, as CIS is concerned with interpretation rather than exhaustive summary, we used information relating to the ‘thickness’ of studies, to inform purposive sampling of studies for analysis from those that met the inclusion criteria. Using NVivo [41], HF first coded fragments of text pertinent to our original research aim from a random selection (n = 12) of publications that had mostly been recorded as having thick description of marketing (11 thick, 1 thin). A diary was maintained alongside coding to help inform interpretation. HF, MW and JA discussed a subsample of codes, prompting refinement and initialising the development of synthetic constructs. Revised codes were reapplied to the initial sample of texts (n = 12), in addition to a further random sample (n = 8). Doing so elaborated the refined codes and synthetic constructs, which were again discussed between HF, MW and JA, this time to seek and interpret relationships between synthetic constructs to develop a synthesising argument. Further thin texts were purposively selected by HF (n = 4), on the basis that they might test or elaborate codes and synthetic constructs, until no new codes were generated (total number of coded texts = 24). The synthesising argument was further refined by HF in consultation with MW and JA. Illustrative samples of texts are used to present the synthesising argument.

### Step (6): consulting with key stakeholders

Finally, as elsewhere [42], we conducted an adapted version of step [6], by inviting informal feedback from other colleagues researching the commercial food system at research group seminars. This led to further refinement of the final synthesising argument and its presentation.

## Results

### Summary of included studies

**Fig. 1:**
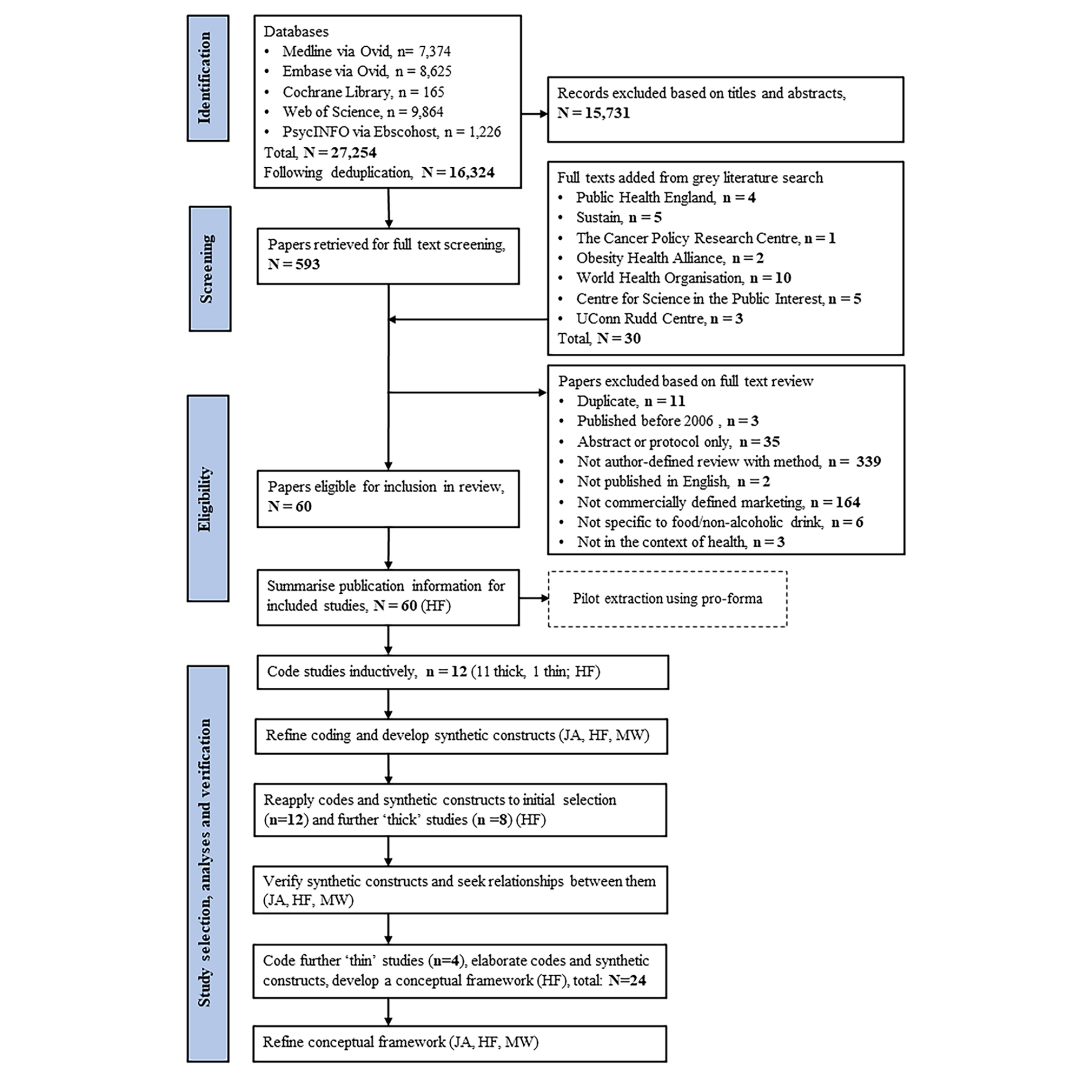
PRISMA diagram with adaptation for critical interpretive synthesis

The search and selection process are summarised in Fig. 1. Following the removal of duplicates, 16,324 titles and abstracts were screened and 623 full texts retrieved. In total, 60 publications met the inclusion criteria (see Additional File 3).

Metadata for the 24 publications selected for synthesis are provided in Additional File 4. Articles were published between 2008 and 2020 and ranged from those studying singular components of marketing (such as price promotions) to the whole marketing mix. Of the initial selection of 12 publications, 11 were classified as thick [43–53], and one as thin [54]. Of the subsequent texts, 8 were thick [55–62] and 4 were thin [63–66]. Of the 24 publications, 4 did not report a conflicts of interest statement [49, 50, 52, 62], and one reported a conflict of interest in an erratum in 2019 [51].

Our synthesis produced a critique of the approach to studying marketing in the public health literature that anchors on the concept of integrated marketing. Example of first, second, and third order constructs (synthetic constructs) that contributed to the development of the synthesising argument are included in Additional File 5.

### Synthesising argument: critique of approach to integrated marketing

While definitions of specific components of marketing, such as price promotions, were more likely to be consistent across publications; marketing as an overarching concept was less consistently defined. Indeed, a definition or framework for marketing, or the specific form of marketing under study, was not present in all publications. While the marketing mix or four Ps (of product, price, promotion and placement) was the most cited conceptual framework, there were differences in how individual components of the marketing mix were defined and understood, particularly promotion.

For example, the definition provided by Grier and Kumanyika appears to reflect the full marketing mix:

*“Promotion includes advertising and other types of persuasive communications that convey product benefits, pricing strategies, and availability (e.g., sales promotions, direct mail, promotional Web sites, public relations, free food samples, special events, and product placement). Place refers to the distribution of the product, including how products are made accessible to target consumers and the quality and convenience of the available products. Price refers to the cost that is exchanged for the product, in absolute terms and relative to alternatives”.*[55, p1617]

In contrast, Blake and colleagues provide a narrower set of examples and include price promotions as part of price, rather than promotion:

*“product (availability and reformulation), price (including price promotions), place, and promotion (including advertising and labelling), or any combination of these”*[63, p1388].

The concept of promotion thus appeared to range from referring to advertising alone, to any activity that exploits prices, product, or placement to encourage a positive perception of a product in the eyes of prospective consumers. Similarly, place was often either explicitly defined or conceived as placement but was considered to encompass a wider set of activities by some authors:

*“place is often misinterpreted as the location of marketing messages, which is in fact a component of promotion. A more accurate definition of place, from a marketing perspective, is the location where behaviours are performed or related goods and services are acquired”.* [58, p275]

Despite differences in explicit conceptualisation, some authors acknowledged that the marketing component under focus in their review might interact with others, indicating that they understood marketing activities to be integrated. In addition to identifying the need for more research that explores the nature and effect of multiple marketing components working together [63], authors also highlighted the disparity in available evidence for different marketing components:

“*While similar marketing techniques may be found across different “media”, those media are most certainly not the same, and some communications platforms are far more studied, understood and evoked than others”* [47, p1].

It was difficult to infer that authors fully understood marketing as taking effect through integrated activities in publications that focused on single aspects of marketing and omitted reference to other components not studied. In articles where authors seemingly moved between concepts without explanation, it is possible that that authors were unclear of the distinction and were using apparently distinct terms synonymously. Such movement included introducing the concept of marketing but going on to draw on evidence only from specific components, such as advertising, without explanation; or interpreting study findings in reference to the wider phenomenon of marketing despite review aims and inclusion criteria taking a narrower focus [51].

Though not the subject of any of the reviews included in the synthesis, strategy was frequently referred to, and may serve as a higher-order mechanism through which separate marketing components are understood to take effect. However, the conceptualisation of strategy was also contested. In some publications, strategy was used to describe a lower-order activity, possibly in only one component of the marketing mix. For example, here the term was used to refer to specific types of activity:

*“multiple marketing strategies or techniques (from spokes-characters, premium offers and health/nutrition related claims to emotional appeals and themes of fun or taste)”.* [47, p1]

Elsewhere, ‘strategy’ was used to mean instances where more than one component of the marketing mix had been used together:

*“reported by strategy type according to the 4Ps of merchandising (product, price, place, and promotion), or reported as a “combined” strategy where more than one of the 4Ps was used at once”* [63, p1388].

Lastly, in some publications, ‘strategy’ appeared to mean a cross-component process that was less easily defined using the 4Ps conceptual framework:

*“creative, sophisticated, and stimulating marketing strategies to produce attractive and engaging content, with audience participation and brand immersion at the forefront of activities”.* [57, p27]

Strategy being harder to articulate, and it being devised by companies ‘behind the scenes’, might be reasons for it not being the core focus of any included reviews.

It was also difficult to find a precise definition or conceptualisation of techniques, tactics or appeal in the sampled literature, which occasionally overlapped with authors’ use of strategy, as above. These activities appeared to cross media and marketing components, often describing specific, creative activities [57, p109]. It was proposed that these terms might refer to processes that increase the cohesion of marketing activities:

*“Brand mascots are used by food and restaurant companies to create a product identity, promote brand personality and continuity across integrated marketing communications”.* [57, p109].

In this sense, these terms seemed to encompass an overarching message that drove specific activities, which was described to take effect by initiating emotional and cognitive responses [60, p30].

Marketing frequently featured alongside other similar concepts, particularly the ‘food environment’. Sometimes, the two concepts explicitly overlapped and were conceptualised as the “marketing environment” [57]. On other occasions it was unclear where authors’ saw the concept of marketing ending and that of the food environment beginning, especially when the definition of the food environment appeared similar to the marketing mix:

*“The food environment has been conceptualized by Glanz et al. as including four aspects*: (1) *the community nutrition environment (e.g., type and location of food outlets);* (2) *the consumer nutrition environment (e.g., availability of healthy food options);* (3) *the organizational nutrition environment (e.g., food access in settings such as schools) and;* (4) *the information environment (e.g., food marketing and advertising)”.* [66, p2]

That marketing takes effect through the integration of multiple actions was indirectly acknowledged when authors described, implicitly or explicitly, the “balloon effect” of regulation. Authors suggested that ignoring one component of marketing – usually the one under study – may not be an effective way of regulating marketing. While most authors recognising a balloon effect suggested that regulation should extend across the 4Ps [58], one proposed that it should also encompass non-market activities:

*“creative, sophisticated, and stimulating marketing strategies to produce attractive and engaging content, with audience participation and brand immersion at the forefront of activities”.* [57, p27]

Inconsistent conceptualisation of integrated marketing extended to the measurement of marketing components. Authors described how use of different research methods, contexts, and locations made it difficult to synthesise results across studies. Exposure to marketing was thought to be particularly difficult to measure, especially through use of an artificially laboratory environment where it was considered impossible to assess the cumulative and longer-term nature of exposure [49].

There was also diversity in the terminology used to search the literature across the texts included, which could allude to different conceptualisations of marketing. For example, in some texts it appeared that a narrow range of search terms were used that orientated around a narrow interpretation of the 4Ps (marketing OR advertising OR promotion [49]), whereas other studies employed a specific search relating to the subset of marketing under study (brand mascot OR character OR cartoon OR licensed [57]).

## Discussion

### Summary of main findings

Using a review of reviews and principles of CIS, we found substantial variation in conceptualisation and operationalisation of many marketing-related terms and in the understanding of food marketing as a strategically integrated activity in the public health literature. Whilst reference to the four Ps framework was common, aspects of this were understood differently by different authors and perhaps not always understood to be constituent parts of a wider, integrated, whole.

### Strength and limitations

Applying principles of CIS enabled us to question epistemological and normative assumptions of the literature – in other words, implicit assumptions contained within the literature – which was particularly advantageous for our research aim compared with other review methods [21]. Inevitably, we could only assess a subset of the sizable literature relevant to our aims. As reviews are purposefully more expansive than singular studies, and may be considered more robust sources of evidence [67], they may be more likely to explore multiple marketing components – and thus, integrated marketing – than primary studies. However, it is possible that marketing is conceptualised and operationalised differently in reviews than in primary studies and similar work focusing on primary studies would have arrived at different findings. Further, our analysis relied on what authors reported in reviews and this may not reflect the totality of how they conceptualise food marketing.

We improved rigour by simultaneous independent screening at both title and abstract, and full text stages. This was preceded by a piloting phase where inclusion criteria were refined and shared understanding developed amongst all reviewers. In addition, repeated discussion of emerging findings during steps 3–5 within the research team, and of near-final results with a wider research group in step 6 ensured the findings were defensible. Nevertheless, how we searched, screened, sampled, and coded the literature was shaped by our own perceptions of marketing and other researchers may have made different decisions leading to different findings. For example, a different search strategy that included “Food” and “Beverages” (MeSH) or “Food” (Emtree) terms may have identified literature that could alter the focus of the synthesis. Though CIS results are inherently influenced by researchers’ views [21] – and in this review were affected by authors’ implicit or explicit understanding of marketing concepts – using an expansive definition of marketing to underpin our work [3], and triangulating screening and interpretation across researchers with different expertise helped to reduce confirmation bias [68]. Whilst we sampled included reviews for synthesis until no new codes were generated, it is difficult to confirm this point had been reached, so we may have inadvertently excluded important additional information in unsampled records.

### Comparison to existing literature

There are several frameworks of food-related marketing literature that aim to partially, or fully, depict the commercial marketing process (e.g. 49, 69). We found many suggestions for improving the evidence base on food marketing in public health research in included reviews, such as calls to measure marketing features more consistently [63], pay more attention to under-examined areas of marketing (e.g. novel digital forms) [60], and improve conceptual clarity [58]. Our synthesising argument goes further by critiquing underlying assumptions of the evidence base to find a broader trend – in the understanding of integrated marketing – in the public health literature. Though frameworks like the four (five, six or seven) Ps are useful for communicating the scope of marketing, our findings indicate that they do not necessarily help develop shared understanding of how concepts are defined and related to each other. We are not aware of a comparable critique of the field.

### Interpretation and implications

Our finding of variation in expressed understanding of marketing as a strategically integrated activity may reflect pragmatic constraints. Tight word limits or disciplinary perspectives in academic journals may constrain authors from expansively discussing their personal conceptualisation of food marketing.

It is also likely that there are differences in how knowledge about food marketing is developed by those working in the marketing and business spheres (through real-world experience and tacit knowledge), and those working in public health research (by developing hypotheses, evidence and theory). This might explain why creative processes identified in our synthesising argument, such as techniques and tactics, were inconsistently conceived. It also emphasises the importance of generating evidence to inform policy intended to regulate food marketing using diverse methods and perspectives.

Our analysis found no clear method or approach for synthesising evidence across different components of marketing to understand its overall, integrated effect. Though conceptual frameworks were used by authors to gather evidence on a range of forms of marketing, there was no evidence of their use to help inform measurement of its integrated effects. The absence of approach to synthesising evidence across multiple components may be because individual components were conceptualised differently making them difficult to synthesise in a review, or because results on individual components are usually presented by authors of primary papers in isolation.

Our findings indicate a possible absence of substantive consideration of exposure to the entirety of integrated food marketing strategies, and the effects of that exposure on health outcomes, in existing literature. For example, whilst reviews aggregate evidence on exposure to e.g. food advertising across different countries [70], there is little aggregate data on exposure to entire marketing campaigns or the effects of different components of these in combination. Greater attention is likely required to developing methods to achieve this, and doing so could help justify more integrated policy responses as seen in Chile. Whilst some notable attempts have been made to capture objective holistic exposure to food marketing, for example, using wearable cameras [71], such studies tend to focus on exposure rather than power, missing part of the key function of marketing [72]. An alternative approach might be to collect objective exposure data (e.g. wearable camera technology [71]) and estimations of power (e.g. through existing coding tools [73]) of a specific campaign. Case study methodology [74] could be used to combine these findings. Such an approach might not achieve the same *breadth* as research that aims to understand exposure to, or power of, a singular marketing activity (e.g. television advertising) across all products, nor could it necessarily attribute causality to a marketing campaign and subsequent eating behaviours. However, it has the potential to achieve *depth* of understanding that better depicts the integrated nature and influence of marketing.

### Unanswered questions and further research

We did not seek to map existing food marketing research, as has been done in some of the included reviews (e.g. 57, 60). Nonetheless, clear opportunities for future research emerged, including a greater focus on understanding the commercial decision-making underpinning food marketing activities, such as marketing strategy. Testing our findings with public health research on other commodities, such a tobacco and alcohol, would help determine whether the patterns we have identified are generalisable. Greater collaboration between public health researchers and marketing researchers in future may also assist with developing a shared conceptual understanding.

Using our analyses, we propose that conceptual clarity and shared understanding of key food marketing terms should help move the public health literature in this field forward. However, such shared understanding may not be achievable. If this is the case, then exploration and to achieve greater understanding of sources of disagreement would, in itself, be useful.

We set out with the broad aim to explore conceptualisation and operationalisation of marketing in public health research, yet the findings produced by our inductive analysis address the former more than the latter. Further work to improve the operationalisation of marketing in public health research might include a systematic evaluation of the different measures of exposure and power that have been used in research. Building such research on a theoretical understanding of how marketing takes effect, and underpinning it with our recommendations for conceptual clarity, is more likely to generate meaningful evidence.

Our critique of integrated marketing builds on the view that marketing takes effect through multiple, concurrent activities. Though this view is widely accepted in business literature [3] and the real-world practice of marketing, it is important to acknowledge that ‘integrated marketing communications’ is a distinct marketing concept (see Table 1), that itself has been implemented differently by marketing practitioners and academics [31]. Furthermore, the distinction between market and other commercial but non-marketing activities (such as lobbying) are increasingly blurred [75], and non-market behaviours might have a growing role in determining consumer behaviour. These trends suggest it may be important to develop our findings with research that draws explicit comparisons between the conceptualisation and operationalisation of marketing among professional marketers and relevant policymakers. This might consist of interviewing members of industry or marketing professionals, or document analysis of publications produced by marketers (e.g. marketing strategy documents).

## Conclusions

Improving the study of food marketing in public health research through better conceptual clarity has the potential to inform policy that is more reflective of the true nature of marketing, and thus more effective in combating food marketing’s effects and protecting public health. Through a focused examination of existing reviews of food marketing in public health research, we found this might be achieved by improving the operationalisation of integrated marketing. Understanding marketing as an integrated set of activities, rather than studying, and regulating, individual marketing components such as advertising, has the potential to lead to more nuanced understanding of how food marketing impacts on public health and, by extension, more effective public health policies.

## Electronic supplementary material

Below is the link to the electronic supplementary material.

Supplementary Material 1

Supplementary Material 2

Supplementary Material 3

Supplementary Material 4

Supplementary Material 5

## Data Availability

The datasets used and/or analysed during the current study are available from the corresponding author on reasonable request.

## Declarations

### Authors’ information

HF is currently affiliated to the Nuffield Department of Primary Care, the University of Oxford, but was affiliated to the MRC Epidemiology Unit, the University of Cambridge, during the conduct of this study. YCU is currently affiliated to the MRC Epidemiology Unit, the University of Cambridge, but was affiliated to Bristol Medical School, the University of Bristol, during the conduct of this study. PAK is currently affiliated to the Baylor College of Medicine, but was affiliated to the University of Cambridge School of Clinical Medicine during the conduct of this study. VT is currently affiliated to the Department of Health and Social Care, but was affiliated to Public Health England during the conduct of this study.

## Abbreviations

NCD: Non-communicable disease
HFSS: High fat, salt and sugar
CIS: Critical Interpretive Synthesis
