# Supplementary material for: The conceptualisation and operationalisation of ‘marketing’ in public health research: a review of reviews focused on food marketing using principles from critical interpretive synthesis
Source: BMC Public Health. 2023 Jul 24;23:1419. doi: 10.1186/s12889-023-16293-4 (PMC10367353; doi:10.1186/s12889-023-16293-4)
Supplement: Supplementary file 2 — Supplementary Material 2 [file 12889_2023_16293_MOESM2_ESM.docx]

## Additional File 2: Database search strategies

**Embase via Ovid**

| 1 | Marketing | (marketing OR sponsorship OR advert* OR celebrit* OR promotion* OR campaign* OR brand* OR logo* OR price* OR pricing OR placement OR persua* OR “social media” OR TV OR televis* OR billboard* OR radio OR magazine* OR newspaper* OR tweet* OR Twitter OR Facebook OR Instagram OR Snapchat OR YouTube OR influencer* OR TikTok OR “Tik Tok” OR label* OR front-of-pack).ab,ti.  Or marketing/ or advertising/ or food labelling/ |
| --- | --- | --- |
| 2 | Food or drink related | (food* or diet or calor* or snack* or eat* or drink* or drunk* or consum* or intak*).ab,ti.  Or food intake/ |
| 3 | Health-related | (unhealth* or health*).ab,ti.  Or health/ |
| 4 | Review | (review).ab,ti.  Or “review”/ |
| 5 | 1 AND 2 AND 3 AND 4 | |
| 6 | Other filters | Limit to English language and 2006 onwards |

**Medline via Ovid**

| 1 | Marketing | (marketing OR sponsorship OR advert* OR celebrit* OR promotion* OR campaign* OR brand* OR logo* OR price* OR pricing OR placement OR persua* OR “social media” OR TV OR televis* OR billboard* OR radio OR magazine* OR newspaper* OR tweet* OR Twitter OR Facebook OR Instagram OR Snapchat OR YouTube OR influencer* OR TikTok OR “Tik Tok” OR label* OR front-of-pack).ab,ti.  OR marketing/ OR Advertising/ OR Food Labeling/ |
| --- | --- | --- |
| 2 | Food or drink related | (food* or diet or calor* or snack* or eat* or drink* or drunk* or consum* or intak*).ab,ti.  OR eating/ OR drinking/ |
| 3 | Health-related | (unhealth* or health*).ab,ti.  or health/ |
| 4 | Review | (review).ab,ti.  OR “review”/ |
| 6 | 1 AND 2 AND 3 AND 4 AND 5 | |
| 6 | Other filters | Limit to (English language) and 2006 onwards |

**Cochrane Library Reviews**

| 1 | Marketing | (marketing OR sponsorship OR advert* OR celebrit* OR promotion* OR campaign* OR brand* OR logo* OR price* OR pricing OR placement OR persua* OR “social media” OR TV OR televis* OR billboard* OR radio OR magazine* OR newspaper* OR tweet* OR Twitter OR Facebook OR Instagram OR Snapchat OR YouTube OR influencer* OR TikTok OR “Tik Tok” OR label* OR front-of-pack):ti,ab,kw  OR MeSH descriptor: [Advertising] this term only OR MeSH descriptor: [Marketing] this term only OR MeSH descriptor: [Food Labeling] this term only OR MeSH descriptor: [Online Social Networking] this term only |
| --- | --- | --- |
| 2 | Food or drink related | (food* or diet or calor* or snack* or eat* or drink* or drunk* or consum* or intak*):ti,ab,kw  OR MeSH descriptor: [Eating] this term only OR MeSH descriptor: [Drinking] this term only |
| 3 | Health-related | (unhealth* OR health*):ti,ab,kw  OR MeSH descriptor: [Health] |
| 4 | Review | (review):ti,ab,kw  OR MeSH descriptor: [Review] this term only |
| 5 | Other filters | Jan 2006 onwards in Cochrane reviews |
| 6 | 1 AND 2 AND 3 AND 4 | |

**Web of Science Core Collection**

| 1 | Marketing | TS=(marketing OR sponsorship OR advert* OR celebrit* OR promotion* OR campaign* OR brand* OR logo* OR price* OR pricing OR placement OR persua* OR “social media” OR TV OR televis* OR billboard* OR radio OR magazine* OR newspaper* OR tweet* OR Twitter OR Facebook OR Instagram OR Snapchat OR YouTube OR influencer* OR TikTok OR “Tik Tok” OR label* OR front-of-pack) |
| --- | --- | --- |
| 2 | Food or drink related | TS= (food* or diet or calor* or snack* or eat* or drink* or drunk* or consum* or intak*) |
| 3 | Health-related | TS=(unhealth* OR health*) |
| 4 | Review | TS=(review) |
| 5 | 1 AND 2 AND 3 AND 4 | |
| 6 | Other filters | Language:(English) and timespan=2006-2020 |

**APA PsychInfo via Ebscohost**

| 1 | Marketing | AB(marketing OR sponsorship OR advert* OR celebrit* OR promotion* OR campaign* OR brand* OR logo* OR price* OR pricing OR placement OR persua* OR “social media” OR TV OR televis* OR billboard* OR radio OR magazine* OR newspaper* OR tweet* OR Twitter OR Facebook OR Instagram OR Snapchat OR YouTube OR influencer* OR TikTok OR “Tik Tok” OR label* OR front-of-pack)  OR TI(marketing OR sponsorship OR advert* OR celebrit* OR promotion* OR campaign* OR brand* OR logo* OR price* OR pricing OR placement OR persua* OR “social media” OR TV OR televis* OR billboard* OR radio OR magazine* OR newspaper* OR tweet* OR Twitter OR Facebook OR Instagram OR Snapchat OR YouTube OR influencer* OR TikTok OR “Tik Tok” OR label* OR front-of-pack)  OR DE “Marketing” OR DE “Digital Marketing” OR DE “Advertising” |
| --- | --- | --- |
| 2 | Food or drink related | AB(food* or diet or calor* or snack* or eat* or drink* or drunk* or consum* or intak*)  OR TI(food* or diet or calor* or snack* or eat* or drink* or drunk* or consum* or intak*)  OR DE “Food” OR DE "Beverages (Nonalcoholic)" |
| 3 | Health-related | AB(unhealth* OR health*) OR TI(unhealth* OR health*)  OR DE "Health" |
| 4 | Review | AB(review) OR TI(review)  OR DE "Literature Review" OR DE "Systematic Review" |
| 5 | 1 AND 2 AND 3 AND 4 | |
|  | Other filters | Language: English and January 2006 onwards |
