# Supplementary material for: The conceptualisation and operationalisation of ‘marketing’ in public health research: a review of reviews focused on food marketing using principles from critical interpretive synthesis
Source: BMC Public Health. 2023 Jul 24;23:1419. doi: 10.1186/s12889-023-16293-4 (PMC10367353; doi:10.1186/s12889-023-16293-4)
Supplement: Supplementary file 3 — Supplementary Material 3 [file 12889_2023_16293_MOESM3_ESM.docx]

## Additional file 3: Full list of studies eligible for inclusion

| **Lead Author** | **Year** | **Title** |
| --- | --- | --- |
| Adeigbe RT, Baldwin S, Gallion K, et al. | 2015 | Food and Beverage Marketing to Latinos: A Systematic Literature Review |
| Bennett R, Zorbas C, Huse O, et al. | 2020 | Prevalence of healthy and unhealthy food and beverage price promotions and their potential influence on shopper purchasing behaviour: A systematic review of the literature |
| Blake MR, Backholer K, Lancsar E, et al. | 2019 | Investigating business outcomes of healthy food retail strategies: A systematic scoping review |
| Boyland EJ, Nolan S, Kelly B, et al. | 2016 | Advertising as a cue to consume: A systematic review and meta-analysis of the effects of acute exposure to unhealthy food and nonalcoholic beverage advertising on intake in children and adults |
| Buchanan L, Kelly B, Yeatman H, et al. | 2018 | The Effects of Digital Marketing of Unhealthy Commodities on Young People: A Systematic Review |
| Cairns G. | 2019 | A critical review of evidence on the sociocultural impacts of food marketing and policy implications |
| Carter M-A, Edwards R, Signal L, Hoek, J. | 2012 | Availability and marketing of food and beverages to children through sports settings: a systematic review |
| Carter, OBJ. | 2006 | The weighty issue of Australian television food advertising and childhood obesity |
| Castro IA, Majmundar A, Williams CB, Baquero B. | 2018 | Customer Purchase Intentions and Choice in Food Retail Environments: A Scoping Review |
| Chapman K, Kelly B, King L. | 2009 | Using a research framework to identify knowledge gaps in research on food marketing to children in Australia |
| Chemas-Velez MM, Gomez LF, Velasquez A. et al. | 2020 | Scoping review of studies on food marketing in Latin America: Summary of existing evidence and research gaps |
| Critchlow N, Angus K, Stead M, et al. | 2019 | Digital Feast: Navigating a digital marketing mix and the impact on children and young people’s dietary attitudes and behaviours |
| Dixon H, Lee A, Scully, M. | 2019 | Sports Sponsorship as a Cause of Obesity |
| Elliott C, Truman E. | 2020 | The Power of Packaging: A Scoping Review and Assessment of Child-Targeted Food Packaging |
| Elliott, C, Truman E. | 2019 | Measuring the Power of Food Marketing to Children: a Review of Recent Literature |
| Englund TR, Zhou M, Hedrick VE, Kraak VI. | 2020 | How Branded Marketing and Media Campaigns Can Support a Healthy Diet and Food Well-Being for Americans: Evidence for 13 Campaigns in the United States |
| Fernqvist F, Ekelund L. | 2014 | Credence and the effect on consumer liking of food A review |
| Folkvord F, van 't Riet J. | 2018 | The persuasive effect of advergames promoting unhealthy foods among children: A meta-analysis |
| Glanz K, Bader MDM, Iyer S. | 2012 | Retail grocery store marketing strategies and obesity: An integrative review |
| Grier SA, Kumanyika SK. | 2008 | The context for choice: Health implications of targeted food and beverage marketing to African Americans |
| Gustafson A, Hankins S, Jilcott S. | 2012 | Measures of the consumer food store environment: A systematic review of the evidence 2000â€“2011 |
| Hallez L, Qutteina Y, Raedschelders M. et al. | 2020 | That's My Cue to Eat: A Systematic Review of the Persuasiveness of Front-of-Pack Cues on Food Packages for Children vs. Adults |
| Hawkes C. | 2009 | Sales promotions and food consumption |
| Hawkes C. | 2008 | Dietary Implications of Supermarket Development: A Global Perspective |
| Ireland R, Chambers S, Bunn C. | 2019 | Exploring the relationship between Big Food corporations and professional sports clubs: a scoping review |
| Jenkin G, Madhvani N, Signal L, Bowers S. | 2014 | A systematic review of persuasive marketing techniques to promote food to children on television |
| Kaur A, Lewis T, Lipkova V. et al. | 2020 | A systematic review, and meta-analysis, examining the prevalence of price promotions on foods and whether they are more likely to be found on less-healthy foods |
| Kaur A, Scarborough P, Rayner M. | 2017 | A systematic review, and meta-analyses, of the impact of health-related claims on dietary choices |
| Kelly B, Baur LA, Bauman AE, King L. | 2011 | Tobacco and alcohol sponsorship of sporting events provide insights about how food and beverage sponsorship may affect children's health |
| Kelly B, King L, Chapman K, et al. | 2015 | A Hierarchy of Unhealthy Food Promotion Effects: Identifying Methodological Approaches and Knowledge Gaps |
| Kraak VI, Story M. | 2015 | Influence of food companies' brand mascots and entertainment companies' cartoon media characters on children's diet and health: A systematic review and research needs |
| Kraak V, Englund T, Misyak S, Serrano E. | 2017 | Progress Evaluation for the Restaurant Industry Assessed by a Voluntary Marketing-Mix and Choice-Architecture Framework That Offers Strategies to Nudge American Customers toward Healthy Food Environments, 2006-2017 |
| McDermott L, O'Sullivan T, Stead M, Hastings G. | 2006 | International food advertising, pester power and its effects |
| Ni Mhurchu C, Vandevijvere S, Waterlander W. et al. | 2013 | Monitoring the availability of healthy and unhealthy foods and non-alcoholic beverages in community and consumer retail food environments globally |
| Obesity Health Alliance | 2019 | Unhealthy Food Marketing: The Impact on Adults |
| Osei-Assibey G, Dick S, MacDiarmid J. et al. | 2012 | The influence of the food environment on overweight and obesity in young children: A systematic review |
| Perez-Ferrer C, Auchincloss AH, de Menezes MC, et al. | 2019 | The food environment in Latin America: a systematic review with a focus on environments relevant to obesity and related chronic diseases |
| Pitt E, Gallegos D, Comans T, et al. | 2017 | Exploring the influence of local food environments on food behaviours: a systematic review of qualitative literature |
| Pournaghi Azar FP, Mamizadeh M, Nikniaz Z. et al | 2018 | Content Analysis of Advertisements Related to Oral Health in Children: A Systematic Review and Meta-analysis |
| Prowse R. | 2017 | Food marketing to children in Canada: a settings-based scoping review on exposure, power and impact |
| Public Health England | 2015 | Sugar Reduction: The evidence for action Annexe 3: A mixed method review of behaviour changes resulting from marketing strategies targeted at high sugar food and non-alcoholic drink |
| Public Health England | 2019 | Foods and drinks aimed at infants and young children: evidence and opportunities for action: Appendix 2 A rapid scoping review examining the role and impact of commercial baby foods and drinks on the diets of children aged 4-36 months |
| Pulker CE, Thornton LE, Trapp GSA. | 2018 | What is known about consumer nutrition environments in Australia? A scoping review of the literature |
| Qutteina Y, De Backer C, Smits T. | 2019 | Media food marketing and eating outcomes among pre-adolescents and adolescents: A systematic review and meta-analysis |
| Ronit K, Jensen JD. | 2014 | Obesity and industry self-regulation of food and beverage marketing: a literature review |
| Russell SJ, Croker H, Viner RM. | 2019 | The effect of screen advertising on children's dietary intake: A systematic review and meta-analysis |
| Sadeghirad B, Duhaney T, Motaghipisheh S, et al. | 2016 | Influence of unhealthy food and beverage marketing on children's dietary intake and preference: A systematic review and meta-analysis of randomized trials |
| Shaw SC, Ntani G, Baird J, Vogel CA. | 2020 | A systematic review of the influences of food store product placement on dietary-related outcomes |
| Silchenko K, Askegaard S, Cedrola E. | 2020 | Three Decades of Research in Health and Food Marketing: A Systematic Review |
| Skaczkowski G, Durkin S, Kashima Y, Wakefield M. | 2016 | The effect of packaging, branding and labeling on the experience of unhealthy food and drink: A review |
| Smith M, Signal L, Edwards R, Hoek J. | 2017 | Children's and parents' opinions on the sport-related food environment: a systematic review |
| Smith R, Kelly B, Yeatman H, Boyland E. | 2019 | Food marketing influences children's attitudes, preferences and consumption: A systematic critical review |
| Smithers LG, Lynch JW, Merlin T. | 2014 | Industry self-regulation and TV advertising of foods to Australian children |
| Sonntag D, Schneider S, Mdege N, Ali S, Schmidt B. | 2015 | Beyond food promotion: A systematic review on the influence of the food industry on obesity-related dietary behaviour among children |
| Truman E, Elliott C. | 2019 | Identifying food marketing to teenagers: A scoping review |
| Velazquez CE, Black JL Kent MP. | 2017 | Food and beverage marketing in schools: A review of the evidence |
| Villegas-Navas V, Montero-Simo MJ, Araque-Padilla RA. | 2020 | The Effects of Foods Embedded in Entertainment Media on Children's Food Choices and Food Intake: A Systematic Review and Meta-Analyses |
| Vukmirovic M. | 2015 | The effects of food advertising on food-related behaviours and perceptions in adults: A review |
| World Health Organization | 2009 | The extent, nature and effects of food promotion to children: a review of the evidence to December 2008 |
| World Health Organization | 2007 | The extent, nature and effects of food promotion to children [‎electronic resource]‎: a review of the evidence |
