# Supplementary material for: The conceptualisation and operationalisation of ‘marketing’ in public health research: a review of reviews focused on food marketing using principles from critical interpretive synthesis
Source: BMC Public Health. 2023 Jul 24;23:1419. doi: 10.1186/s12889-023-16293-4 (PMC10367353; doi:10.1186/s12889-023-16293-4)
Supplement: Supplementary file 4 — Supplementary Material 4 [file 12889_2023_16293_MOESM4_ESM.docx]

## Additional file 4: List of studies included in synthesis

| **Author(s)** | **Year** | **Type of review** | **Marketing component under study** | **Thick or thin** | **Conflict of interest (CoI) declaration ^i^** |
| --- | --- | --- | --- | --- | --- |
| Adeigbe RT, Baldwin S, Gallion K, et al. (43) | 2015 | Systematic literature review; narrative presentation of results | Product, price, promotion and place | Thick | Yes – no CoI declared |
| Bennett R, Zorbas C, Huse O, et al. (45) | 2020 | Systematic literature review; narrative presentation of results | Price promotions | Thick | Yes – CoI declared |
| Blake MR, Backholer K, Lancsar E, et al. (63) | 2019 | Systematic literature review; narrative presentation of results | Retail strategies | Thin | Yes – source of funding statement |
| Boyland EJ, Nolan S, Kelly B, et al. (46) | 2016 | Systematic review; meta-analysis | Advertising | Thick | Yes – CoI declared |
| Critchlow N, Angus K, Stead M, et al. (60) | 2019 | Literature review; narrative presentation of results | Digital marketing | Thick | **No** |
| Elliott C, Truman E. (47) | 2020 | Systematic scoping review; narrative presentation of results | Packaging | Thick | Yes – no CoI declared |
| Grier SA,  Kumanyika SK. (55) | 2008 | Systematic literature review; narrative presentation of results | Product, price, promotion and place | Thick | Yes – CoI declared |
| Hallez L, Qutteina Y, Raedschelders M, et al. (56) | 2020 | Systematic literature review; narrative presentation of results | Packaging | Thick | Yes – no CoI declared |
| Kaur A, Lewis T, Lipkova V, et al. (48) | 2020 | Systematic literature review; meta-analyses | Price promotions | Thick | Yes – CoI declared |
| Kelly B, King L, Chapman K, et al. (49) | 2015 | Literature review; narrative presentation of results | Food promotion | Thick | **No** |
| Kraak VI, Story M. (57) | 2015 | Systematic literature review; narrative presentation of results | Brand mascots and media characters | Thick | Yes – no CoI declared |
| Ni Mhurchu C, Vandevijvere S, Waterlander W, et al. (54) | 2013 | Systematic literature review; narrative presentation of results | Consumer retail food environments | Thin | Yes – no CoI declared |
| Pournaghi Azar FP, Mamizadeh M, Nikniaz Z, et al. (44) | 2018 | Systematic review; meta-analysis | TV advertisements | Thick | Yes – no CoI declared |
| Prowse R. (58) | 2017 | Systematic scoping review; narrative presentation of results | Exposure to food marketing by setting | Thick | Yes – no CoI declared |
| Public Health England (59) | 2019 | Systematic literature review; narrative presentation of results | Product, price, promotion and place | Thick | Yes – no CoI declared |
| Qutteina Y, De Backer C, Smits T. (50) | 2019 | Systematic literature review; meta-analysis | Media food marketing | Thick | Yes – no CoI declared |
| Sadeghirad B, Duhaney T, Motaghipisheh S, et al. (51) | 2016 | Systematic review; meta-analysis | Marketing overall | Thick | Yes – CoI declared in erratum |
| Silchenko K, Askegaard S, Cedrola E. (52) | 2020 | Systematic literature review; narrative presentation of results | Overall mapping | Thick | **No** |
| Skaczkowski G, Durkin S, Kashima Y, Wakefield M. (64) | 2016 | Literature review; narrative presentation of results | Packaging, branding and labelling | Thin | Yes – no CoI declared |
| Smith M, Signal L, Edwards R, Hoek J.(65) | 2017 | Systematic literature review; narrative presentation of results | Sport related food environment | Thin | Yes – no CoI declared |
| Smithers LG, Lynch JW, Merlin T. (53) | 2014 | Systematic literature review; narrative presentation of results | TV advertising | Thick | Yes – CoI declared |
| Velazquez CE, Black JL, Kent MP. (66) | 2017 | Literature review; narrative presentation of results | Marketing environment in schools | Thin | Yes – no CoI declared |
| Villegas-Navas V, Montero-Simo MJ, Araque-Padilla RA. (61) | 2020 | Systematic literature review; meta-analyses | Media placement | Thick | Yes – no CoI declared |
| Vukmirovic M. (62) | 2015 | Literature review; narrative presentation of results | Advertising | Thick | **No** |

^i^ Accepted information included an explicit conflict of interest declaration or a sources of funding statement related to the food and drinks industry.
