# Supplementary material for: The conceptualisation and operationalisation of ‘marketing’ in public health research: a review of reviews focused on food marketing using principles from critical interpretive synthesis
Source: BMC Public Health. 2023 Jul 24;23:1419. doi: 10.1186/s12889-023-16293-4 (PMC10367353; doi:10.1186/s12889-023-16293-4)
Supplement: Supplementary file 5 — Supplementary Material 5 [file 12889_2023_16293_MOESM5_ESM.docx]

**Appendix 5: Examples of key third, second and first order constructs that underpinned the synthesising argument (critique of approach to integrated marketing)**

| **Example of third order constructs** | **Examples of constituent second order constructs** | **Examples of constituent first order constructs** |
| --- | --- | --- |
| **Balloon effect** | Marketing responds to regulation | "The comparison of the results of these studies showed that although there is a good adherence to these regulations, children are still targeted by marketers in different ways" (44, p115) |
|  |  | "Policy makers may also consider adopting additional supporting interventions that target broader aspects of marketers' 4Ps, such as product availability through industry reformulation, or food pricing via taxes and subsidies, in a whole system intervention to reduce the impact of food marketing" (58, p289) |
|  | Current regulation insufficient | "This report shows the pressing need for regulatory change, across all media platforms" (60, p4) |
| **Pinpointing strategy** | Strategies are specific activities | "food marketing captures...multiple marketing strategies or techniques (from spokes-characters, premium oﬀers and health/nutrition related claims to emotional appeals and themes of fun or taste)" (47, p1) |
|  |  | "Foods embedded in entertainment media might constitute another marketing strategy (in the case of branded foods) or it might not (non-branded foods)" (61, p14) |
|  | Strategy drives activities | "There is also evidence from content analysis research that digital marketing uses a variety of creative, sophisticated, and stimulating marketing strategies to produce attractive and engaging content, with audience participation and brand immersion at the forefront of activities" (60, p27) |
| **Temporality** | Accumulating over time | "The collective effects of continued exposure to food marketing that occurs in real life and over a lifetime may lead to an amplification of these effects, particularly when the marketing is repetitious and delivered over multiple platforms and many settings" (46, p531) |
|  |  | "Although the hypothetical effect model is depicted as a linear progression, it is likely that many of the levels are recurrent and result in positive feedback and reinforcement of earlier levels" (49, p e92) |
|  |  | "The collective effects of continued exposure to food marketing that occurs in real life and over a lifetime may differ" (51, p956) |
|  | Unclear long term effects | [In reference to packaging cues] "Little is known about whether the effect of these cues persists in the long term" (56, p12) |
|  | Short term marketing | "'Price promotion' was defined as a consumer-facing temporary price reduction or discount available to all customers" (48, p1284) |
| **Measuring marketing components** | Cannot capture breadth of marketing in singular studies | "Moreover, because studies have tended to focus on just some marketing elements and are therefore not sufficiently comprehensive, it is difficult to understand the totality of food and beverage marketing that students are exposed to" (66, p10) |
